# Supplementary material for: Constitutive Expression of a Cytotoxic Anticancer Protein in Tumor-Colonizing Bacteria
Source: Cancers (Basel). 2023 Feb 27;15(5):1486. doi: 10.3390/cancers15051486 (PMC10000871; doi:10.3390/cancers15051486)
Supplement: Supplementary file 1 [file cancers-15-01486-s001.zip › cancers-2238189-supplementary.docx]

**Supplemental information**

**Constitutive expression of a cytotoxic anticancer protein in tumor-colonizing bacteria**

Phuong-Thu Mai, Daejin Lim, EunA So, Ha Young Kim, Taner Duysak, Thanh-Quang Tran, Miryoung Song, Jae-Ho Jeong* and Hyon E. Choy*.

**Supplemental materials and methods**

**Promoter activity analysis**

The bacteria carrying the plasmid prrnBP1-gfpOVA were cultured overnight and sub-cultured into 50 mL fresh LB containing ampicillin (100 µg/mL) at a dilution of 1:100. During the culture period, samples were collected for testing optical density at an absorbance of 600 nm using a spectrophotometer (Shimazu, Japan, UV-1800). Bacterial samples (1 mL) were centrifuged, and the pellets were washed with 1× PBS and fixed with 3.9% formaldehyde. Green fluorescent protein (GFP) expression was measured using a fluorometer (Thermo Scientific, VarioskanLux) at λ_excitation_ = 488 nm and λ_emission_ = 525 nm. The fluorescence intensity was normalized by calculating the fraction of fluorescent signals over bacterial cell mass (AU = Fluorescent signals/A_600_).

**Cell viability assay**

ΔppGpp *S*. Gallinarum (SG4023) and ΔppGpp ΔglmS *S.* Gallinarum carrying prrnBP1-psp-TP (SMP4003) were cultured overnight. The overnight cultures were diluted 1:100 in fresh LB medium and grown in a 37°C shaking incubator. After 7 h, 50 mL bacteria were centrifuged at 4000 rpm for 15 min. The supernatant fractions were obtained by the filtration through 0.22 µm-pore-size syringe filter (Merck Millipore, SLGVR33RS) to totally remove bacterial cells. The filtrate of supernatants was concentrated using Centricon columns (Merck Millipore, Amicon Ultra – 15, UFC900308) and the total protein concentrations were examined using Bradford method.^1^ CT26 and 4T1 cells were seeded into 96-well microplates at a density of 1 × 10^4^ cells per well and incubated for 16 h at 37°C in CO_2_ incubator. The cells were treated with the supernatant fractions containing 1 µg of total proteins per well. After 24 h of treatment, 10 µL WST-8 (Biomax, Quanti-Max WST-8 cell viability assay kit, QM1000) was added into the mixtures, and they were incubated for 2 h. The absorbance at 450 nm was determined using a microplate reader (Thermo Scientific, VarioskanLux).


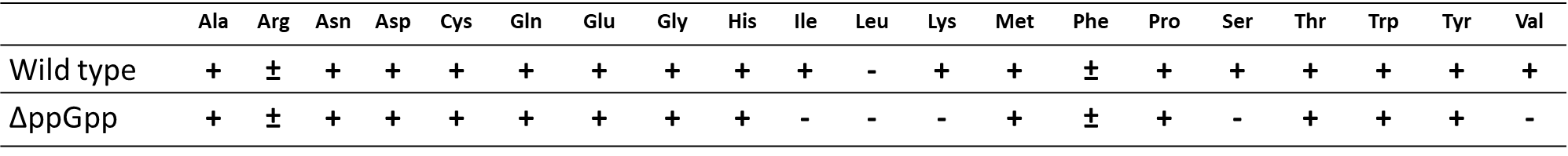


**Table S1. Amino acid requirements of wild-type *S.* Gallinarum and ΔppGpp *S.* Gallinarum**

Amino acid requirements were scored on M9 minimal glucose plates containing combinations of 19 amino acids at 100 µg/mL with 1 of the full set of 20 omitted. +, normal growth; ±, poor growth or microcolonies; -, no visible growth after 24–48 h of incubation.


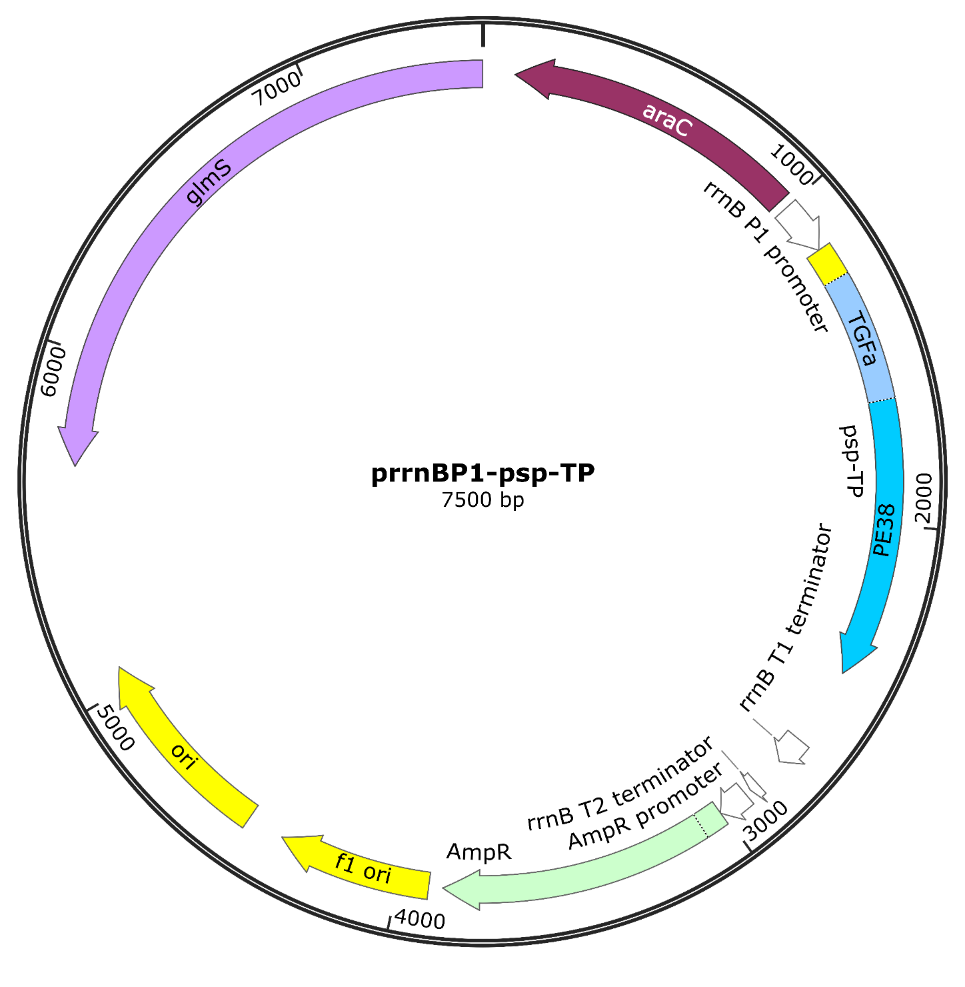


**Figure S1: The plasmid map of the prrnBP1-psp-TP.** This plasmid also contains the *glmS* gene for stable maintenance of plasmid. (psp : MGLKMKKRSGKKAWMLLVMSLLIAAVPITASAA)


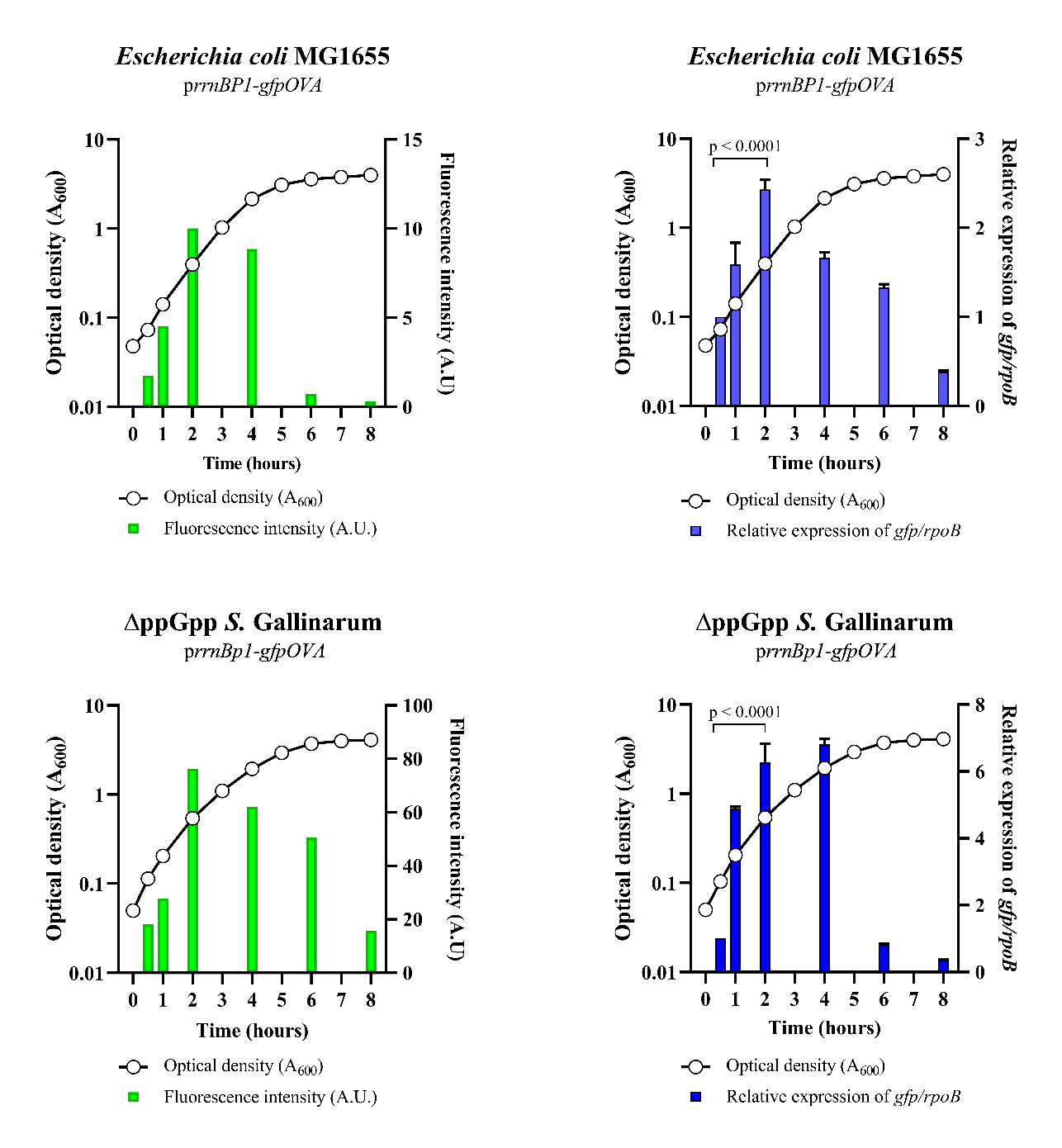


**B**

**A**

**Figure S2. *rrnB P1* promoter activity in *E. coli* K-12 MG1655 and ΔppGpp *S.* Gallinarum *in vitro.***

*E. coli* MG1655 **(A)** and ΔppGpp *S.* Gallinarum **(B)** were transformed with prrnBP1-gfpOVA and grown in LB broth at 37°C with vigorous aeration. At the indicated times, bacterial samples were collected to measure cell mass at A_600_ and fluorescence intensity at 488–525 nm using a fluorometer (left panels). Representative data are the results of three independent replicates. A.U. = fluorescence intensity (488–525 nm)/A_600_. The *rrnB* P1 promoter activity at each time point was examined by quantitative real-time PCR to measure the level of *gfp* mRNA relative to a housekeeping *rpoB* mRNA (right panels). The ΔΔC_t_ values were calculated in triplicate (unpaired Student’s *t*-tests, P < 0.0001).

**DAPI**

**S.G**

**Merge**

2 hours

6 hours

12 hours

72 hours

**Tumor**

**A**


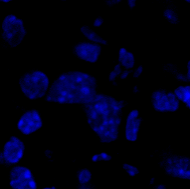

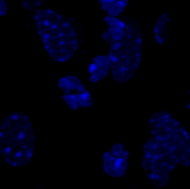

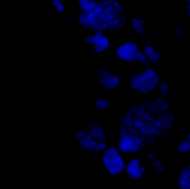

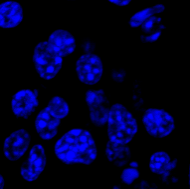


**F4/80**


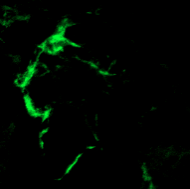

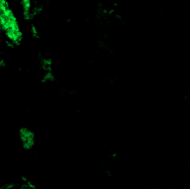

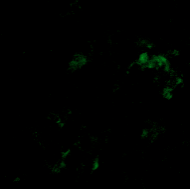

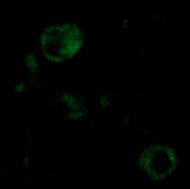


**5μm**


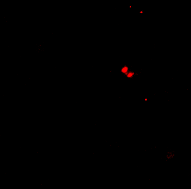

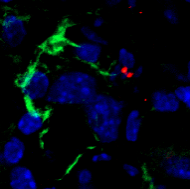

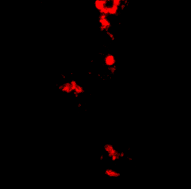

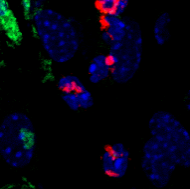

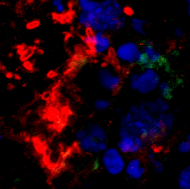

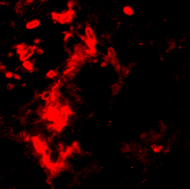

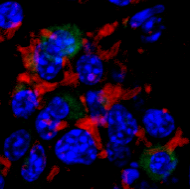

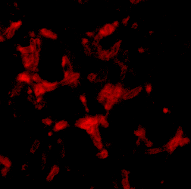


**Spleen**


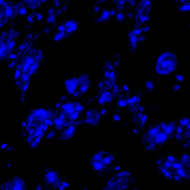

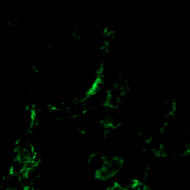

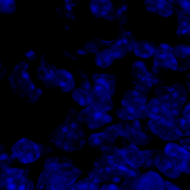

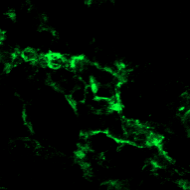

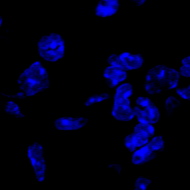

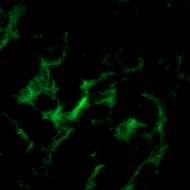

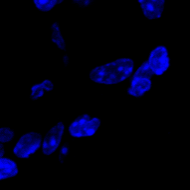

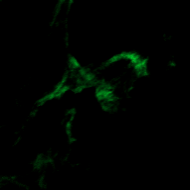


**DAPI**

**S.G**

**Merge**

**F4/80**

2 hours

6 hours

12 hours

72 hours

**5μm**


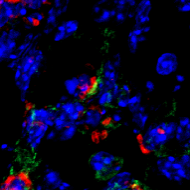

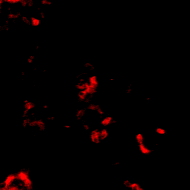

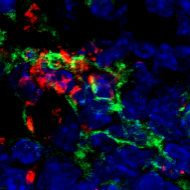

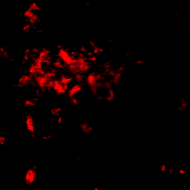

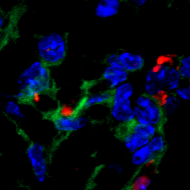

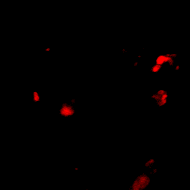

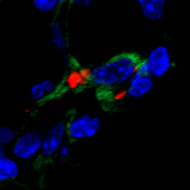

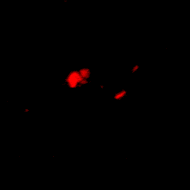


**B**

**Liver**


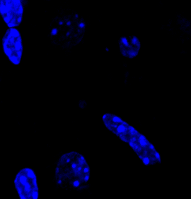

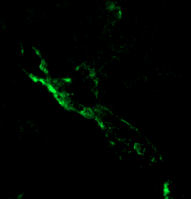

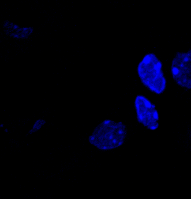

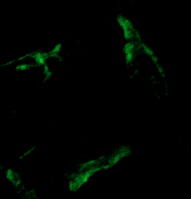

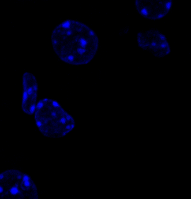

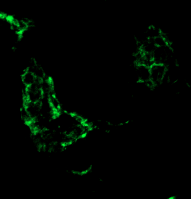

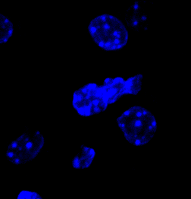

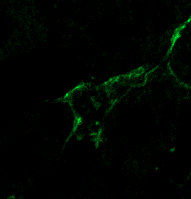


**DAPI**

**S.G**

**Merge**

**F4/80**

2 hours

6 hours

12 hours

72 hours

**5μm**


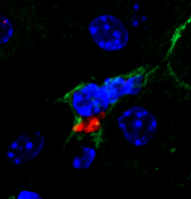

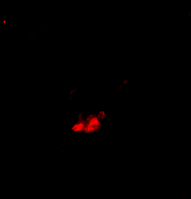

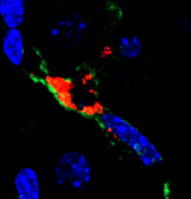

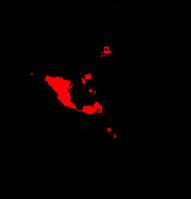

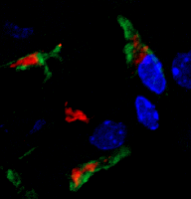

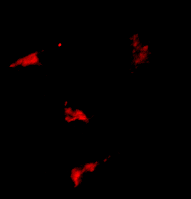

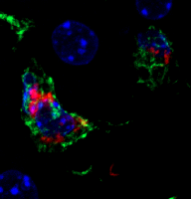

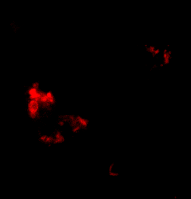


**C**

**D**

**72 hours**

**Tumor**

**Spleen**

**Liver**


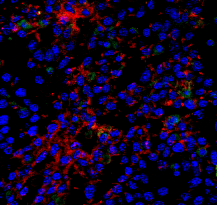

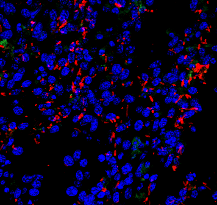

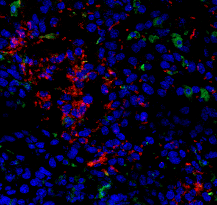

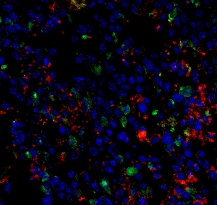

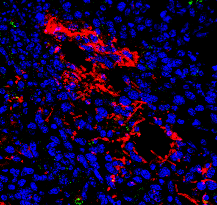

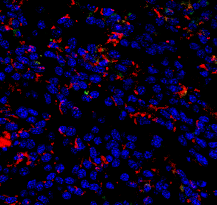

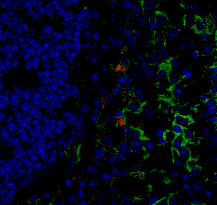

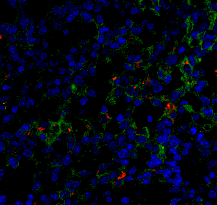

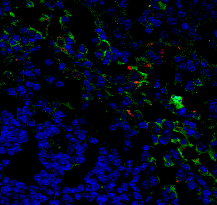

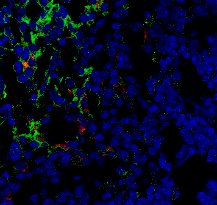

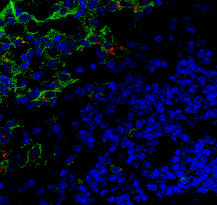

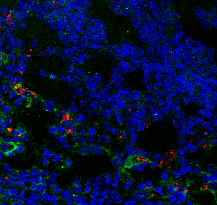

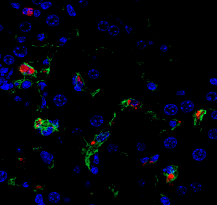

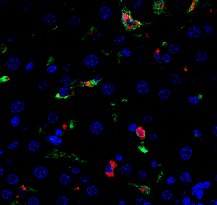

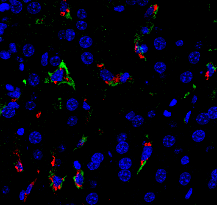

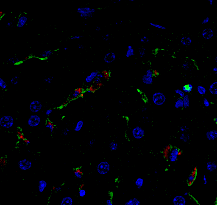

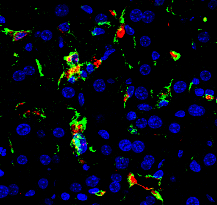

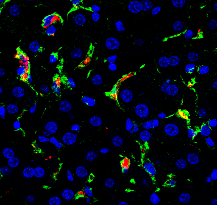


**10μm**

**Figure S3. Co-localization of macrophages and *Salmonella* in tumor tissues and in the RES.**

As shown in the images in Figure 2D, F4/80^+^ macrophages and *Salmonella* were stained with specific antibodies and observed under a confocal microscope (DAPI: nuclei; Alexa Fluor 633-conjugated goat anti-rabbit antibody-stained ΔppGpp *S.* Gallinarum in red; Alexa Fluor 488-conjugated goat anti-rat antibody-stained F4/80^+^ macrophages in green). Scale bar = 5 µm for 1600× magnification (A, B, and C). Scale bar = 10 µm for 400× magnification (D). Imaginary macrophages were drawn with dotted lines.


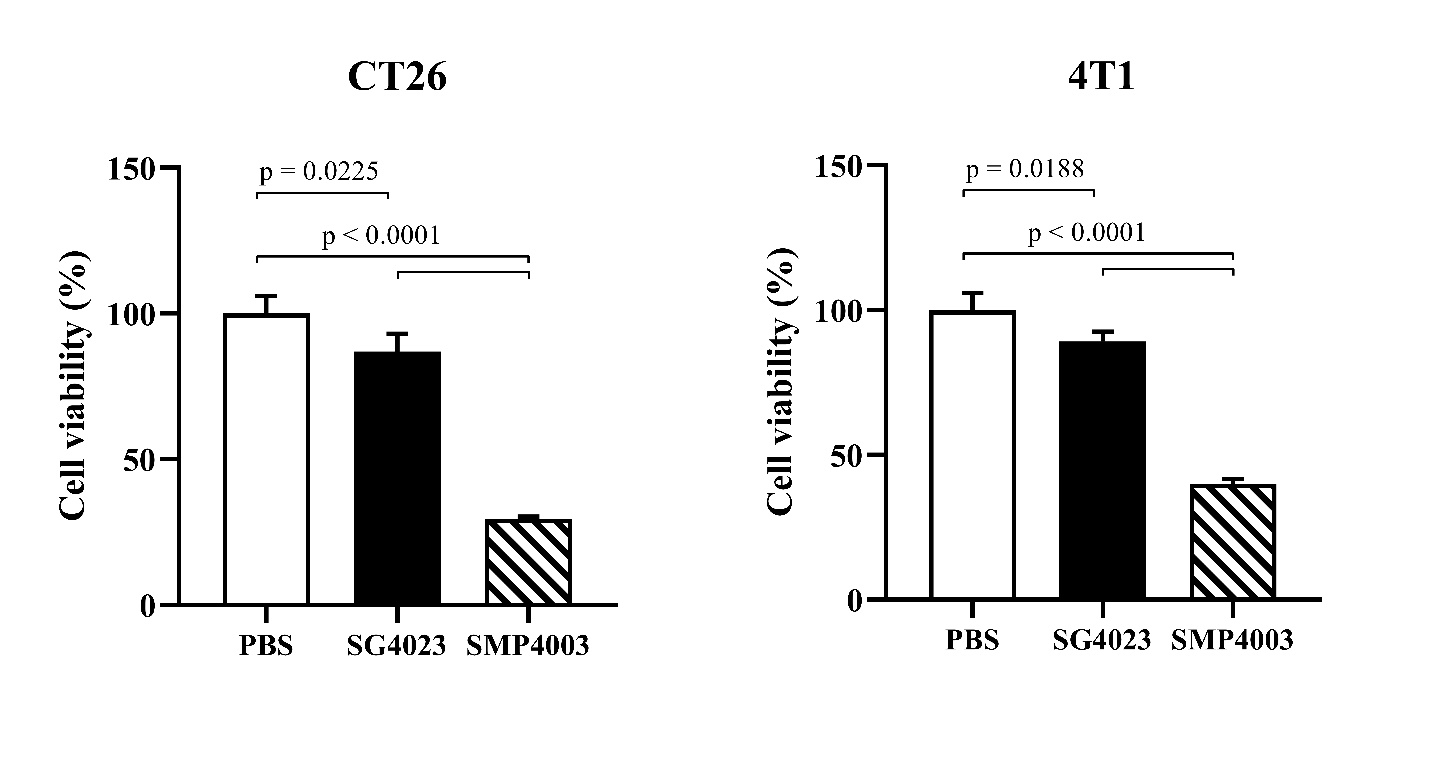


**A**

**B**

**Figure S4. Cancer cell killing by TP released from ΔppGpp *S.* Gallinarum.**

Bacterial supernatants of ΔppGpp *S.* Gallinarum (SG4023) or those carrying the plasmid prrnBP1-psp-TP (SMP4003) were collected when the cultures entered the stationary phase. The proteins were concentrated by centrifugation and filtration. The CT26 and 4T1 cell lines were seeded in 96-well microplates at a density of 10^4^ cells per well. Bacterial supernatants containing 1 µg protein were added to the adherent cells when the cell mass reached approximately 10^5^ cells/well. After 1 day of treatment, 10 µL WST-8 was added and incubated for 2 h. The absorbance of samples was measured at 450 nm with a microplate reader. Data are presented as the mean ± SD (n = 4/group). The significance of differences is indicated by P < 0.05 (unpaired Student’s *t-*tests).

**
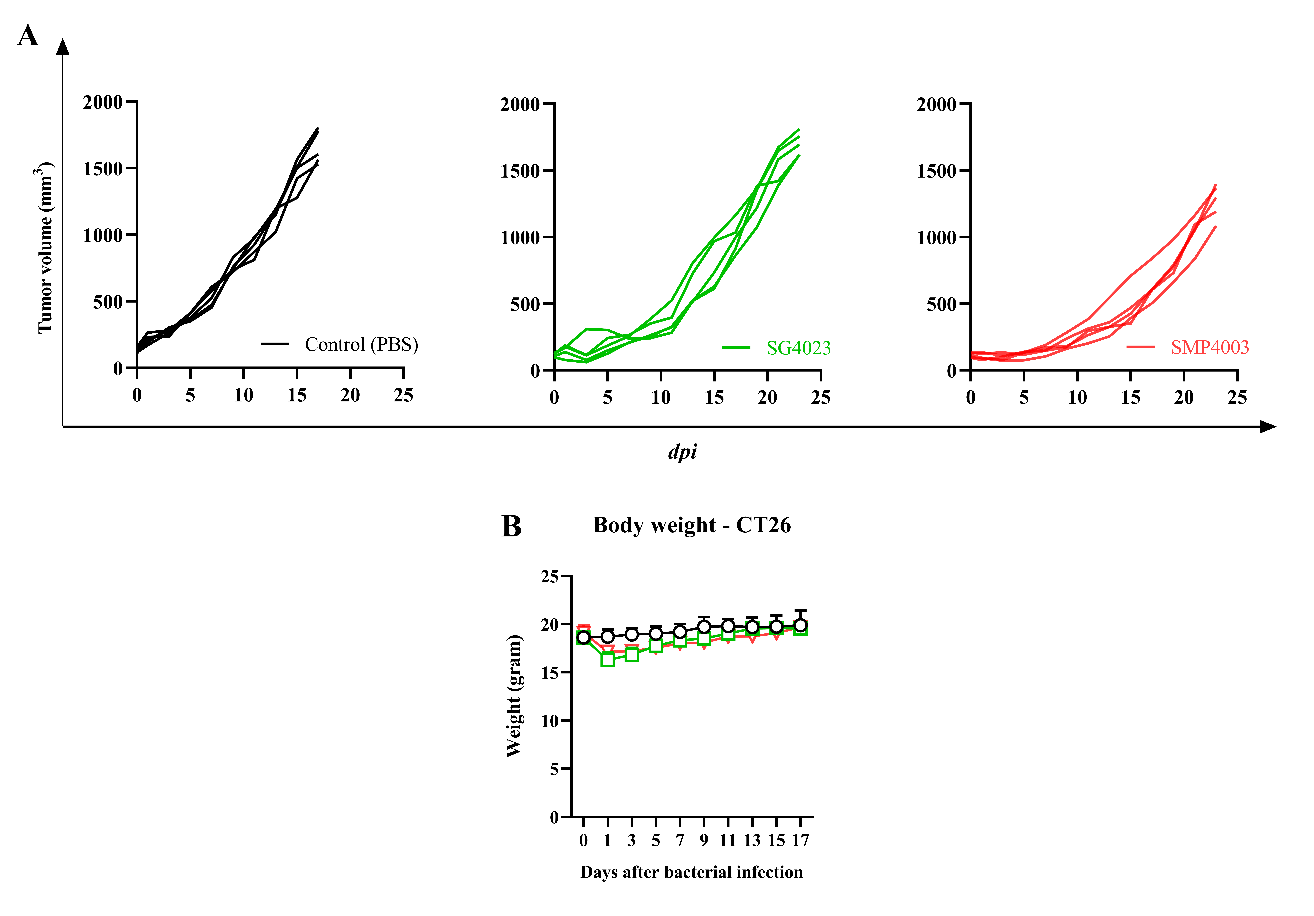
**

**
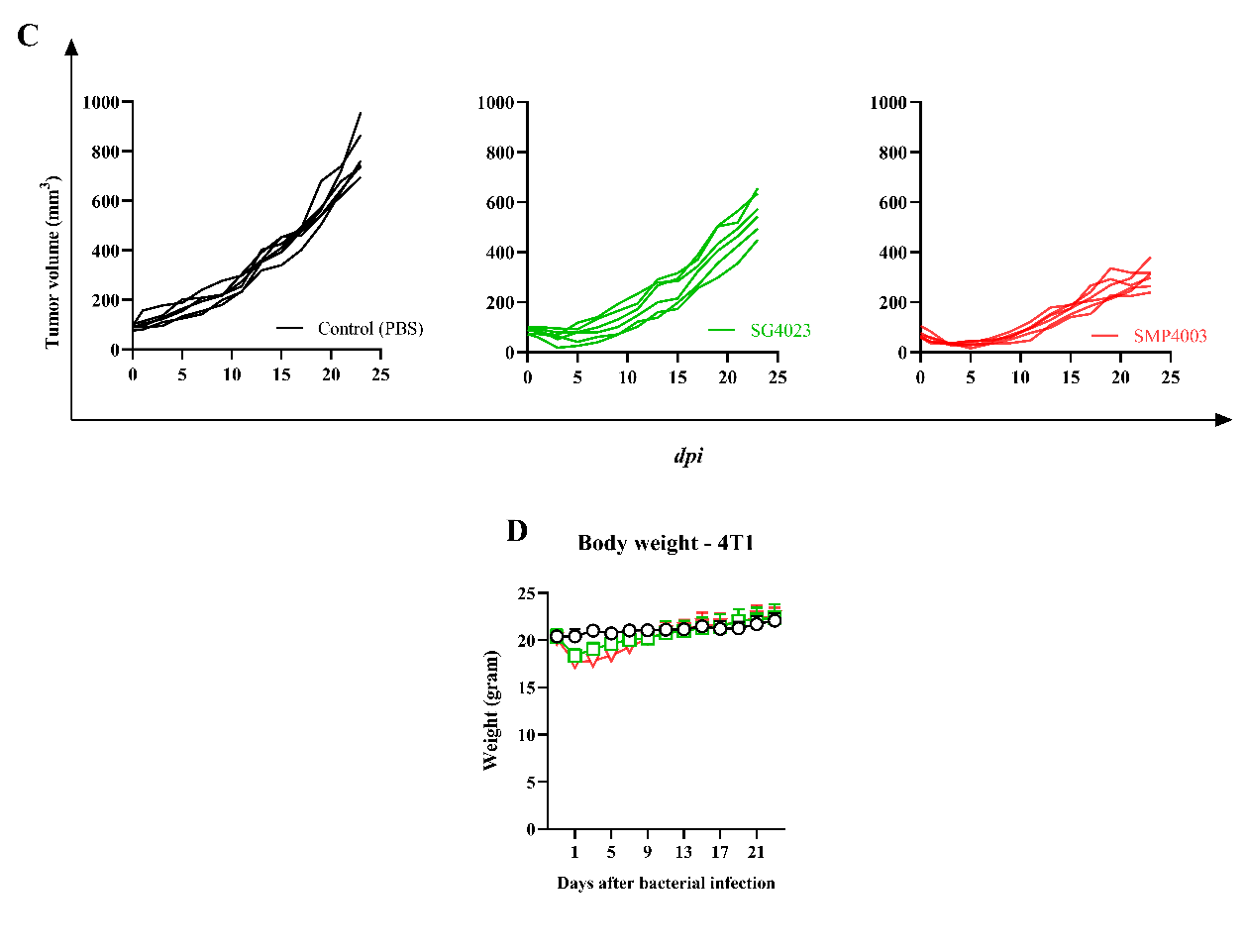
**

**Figure S5. Antitumor effect of ΔppGpp *S.* Gallinarum carrying prrnBP1-psp-TP in BALB/c mice grafted with CT26 colon carcinoma and 4T1 murine breast cancer cells.**

Individual tumor size changes of CT26 (n = 5 mice per group) **(A)** or 4T1 (n = 6 mice per group) **(C)** tumor-grafted mice treated with PBS, ΔppGpp *S.* Gallinarum (SG4023), or ΔppGpp *S.* Gallinarum harboring prrnBP1-psp-TP (SMP4003) (related to the data shown in Figure 4). Effects of bacterial infection on the body weight of mice grafted with CT26 **(B)** or 4T1 **(D)** cells (related to data shown in Figure 4).


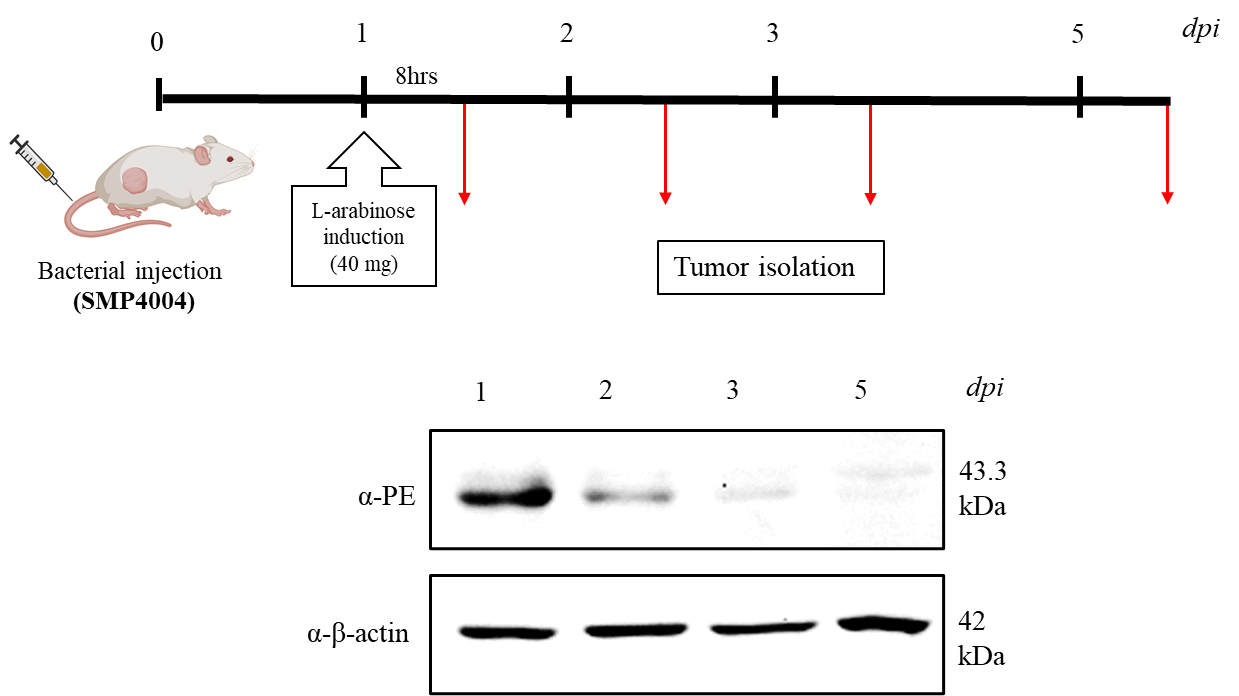


**Figure S6. Secretion of TP under the control of the inducible *araBAD* promoter from ΔppGpp *S.* Gallinarum colonizing CT26 grafted in mice.**

The ΔppGpp *S.* Gallinarum carrying pSEC-TGFα-PE38 (SMP4004) were intravenously injected into mice grafted with CT26 colon cancer cells (1 × 10^8^ CFU/mouse). At 1-day post-injection, 40 mg L-arabinose was injected intraperitoneally into mice. Tumors were isolated at the indicated time points and homogenized in a protein-protected buffer to collect supernatants. TP was detected by western blotting. β-actin was used as the loading control. Representative data are the results of two independent replicates. Uncropped membranes are shown in F and G panels in Figure S7.


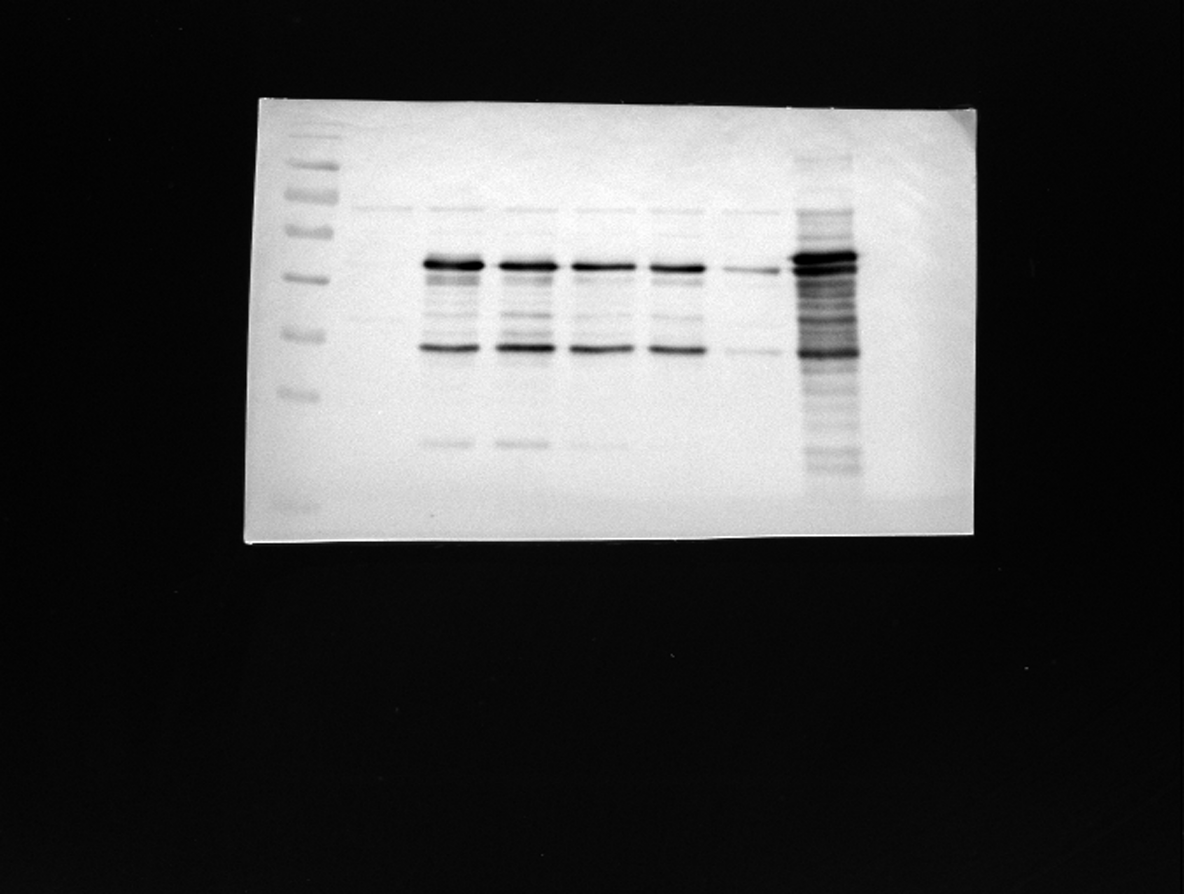

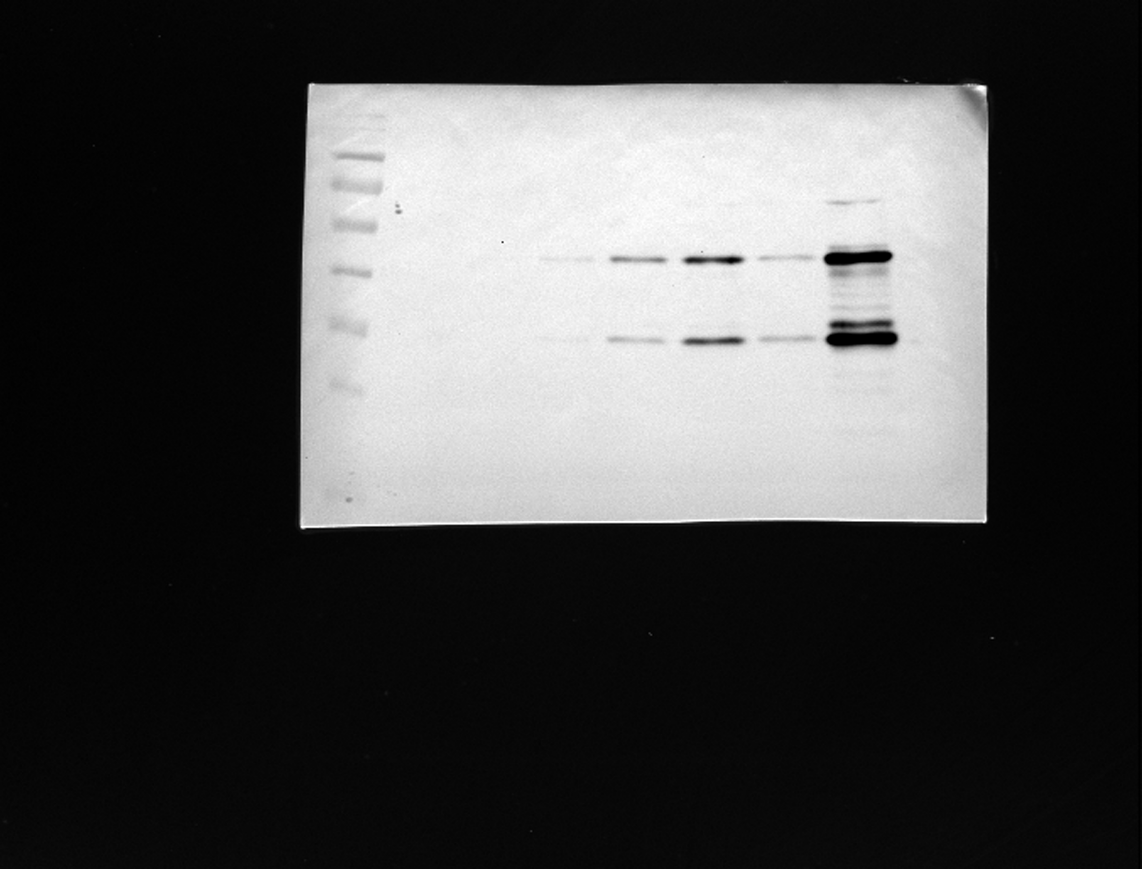


**A**

**B**


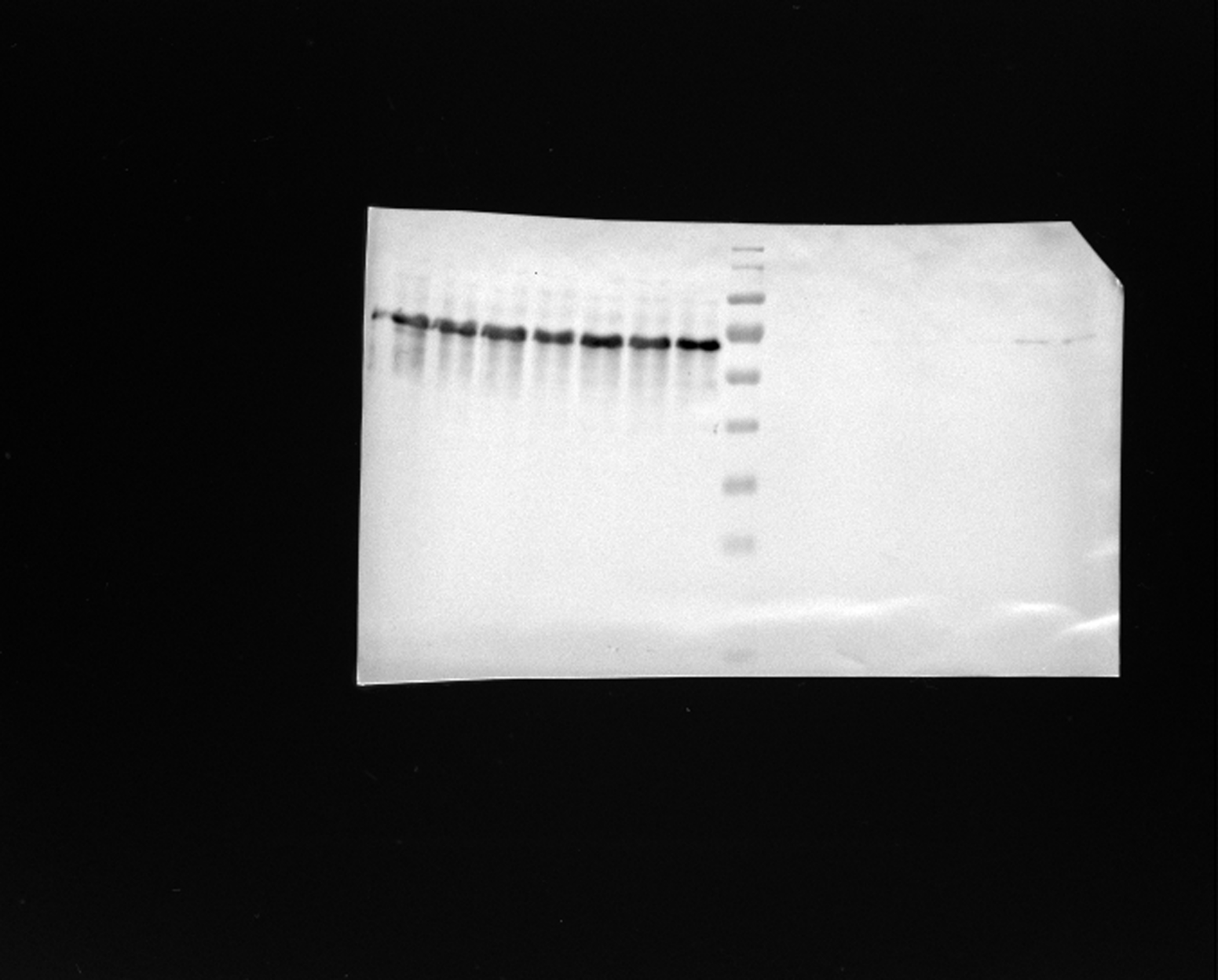


**C**


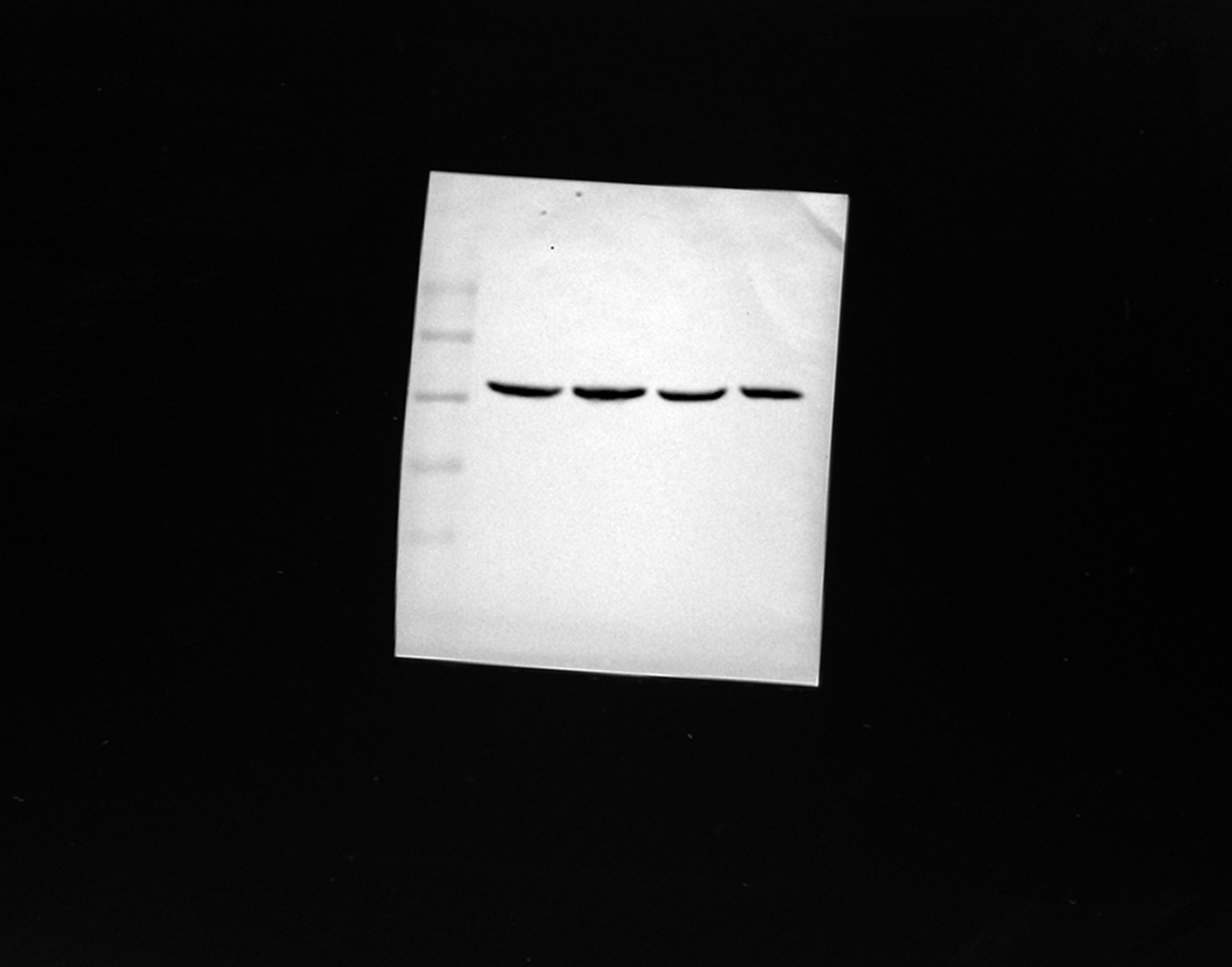

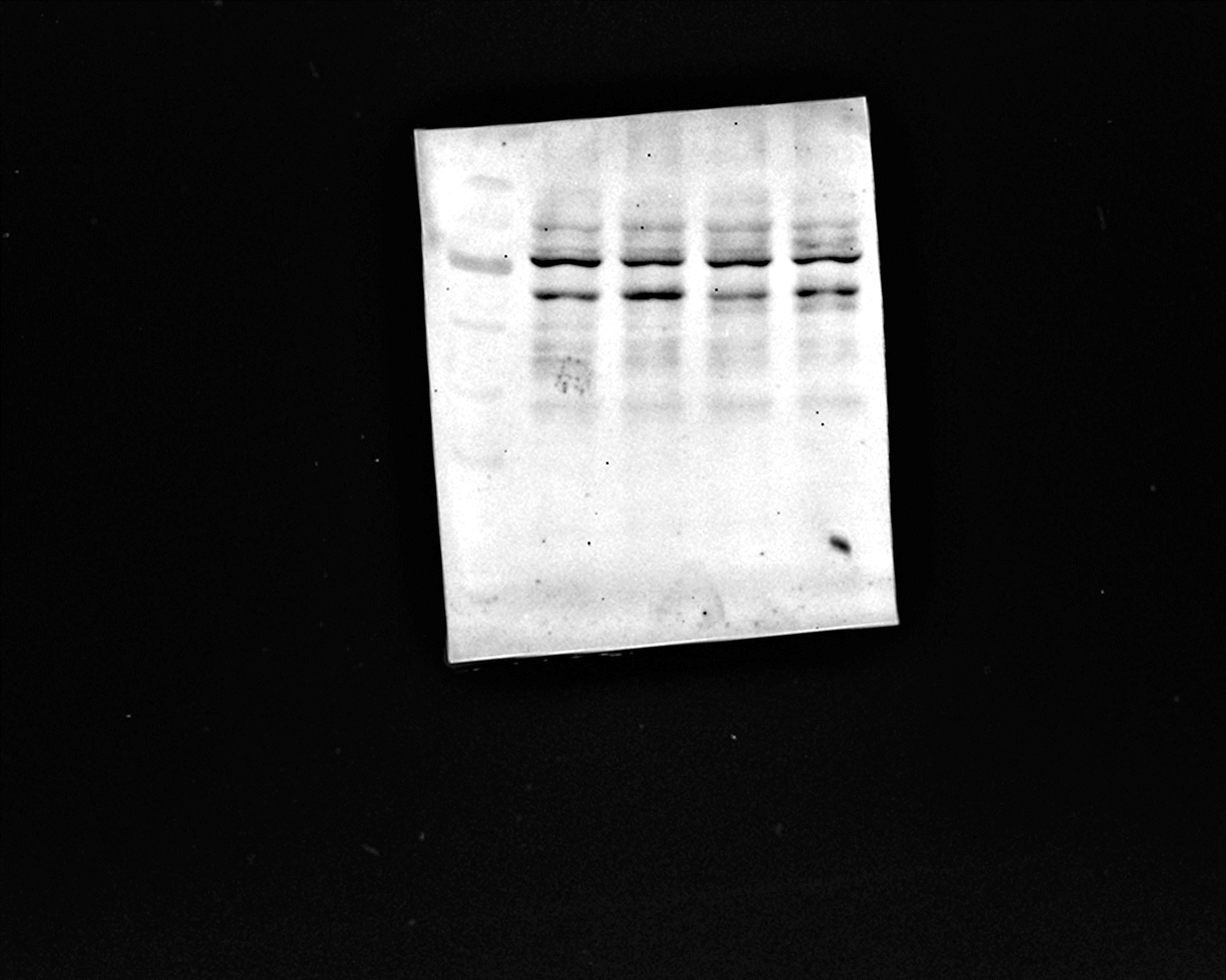


**D**

**E**


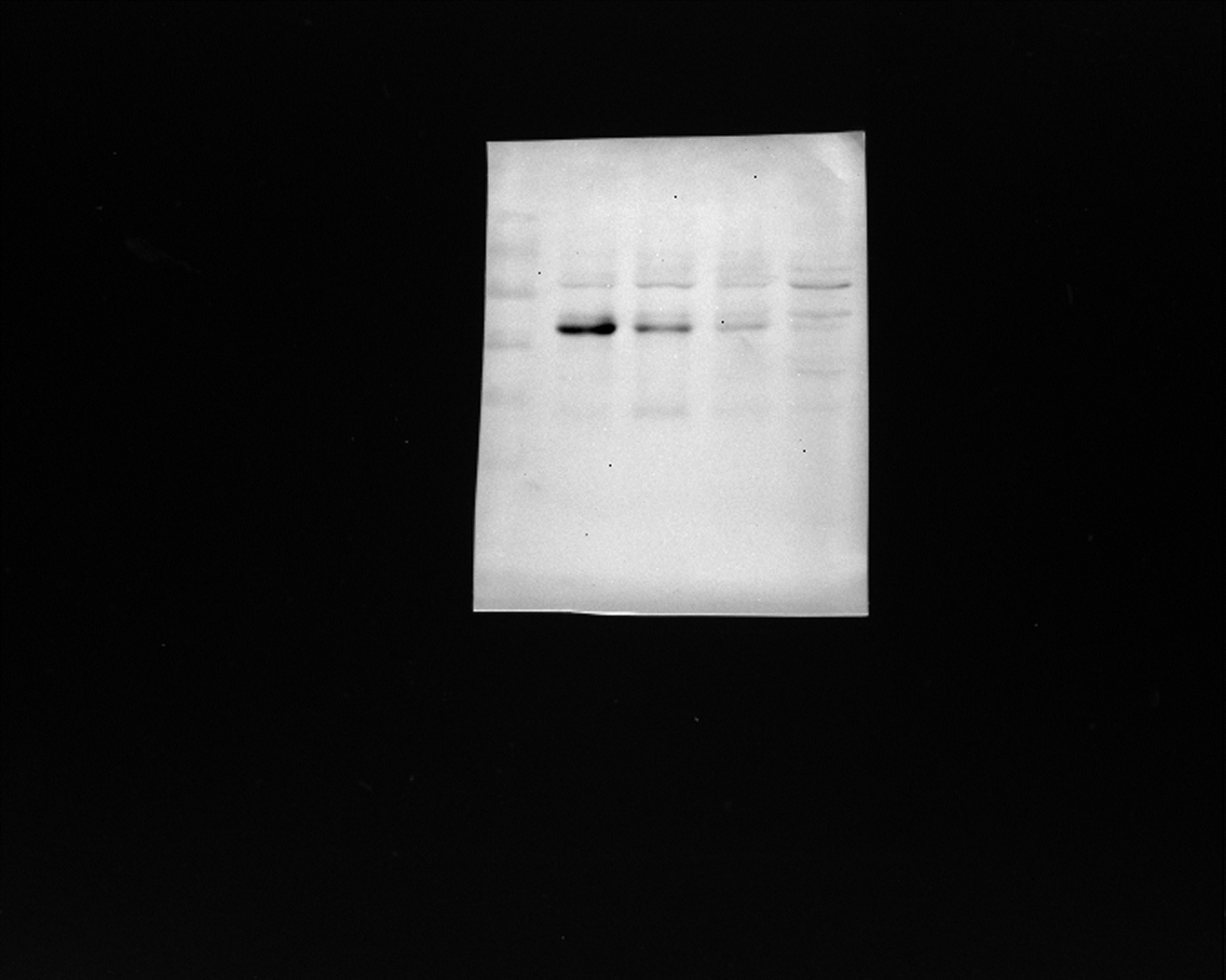

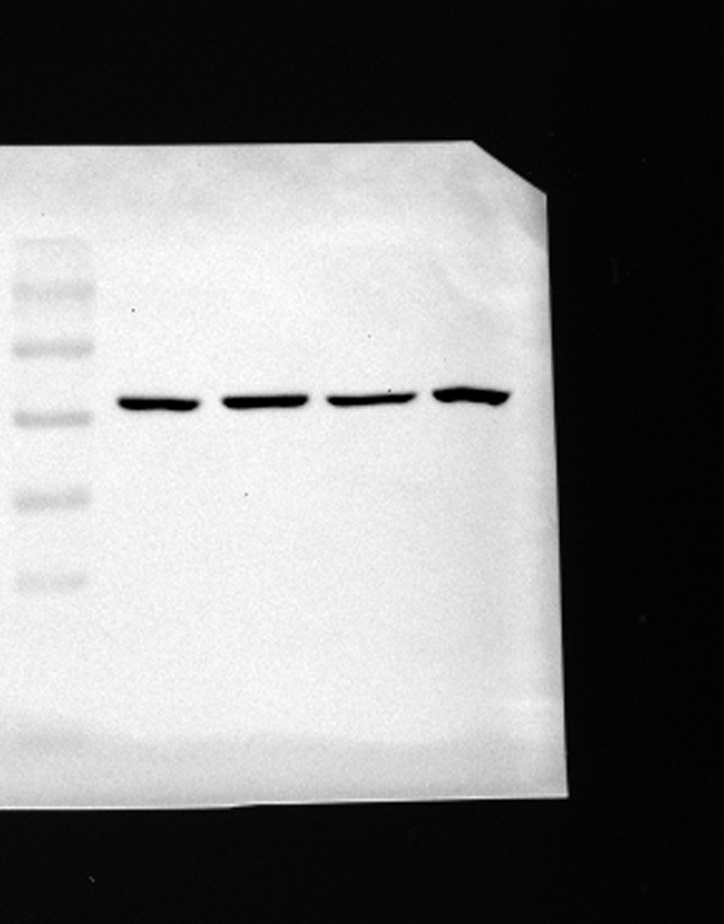


**F**

**G**

**Figure S7. Western blot analysis shown in uncropped membranes.**

Panel A: Anti-PE antibody with pellet (related to data shown in Figure 3A).

Panel B: Anti-PE antibody with supernatant (related to data shown in Figure 3A).

Panel C: Anti-GroEL antibody with pellet and supernatant (related to data shown in Figure 3A).

Panel D: Anti-PE antibody with CT26 tumor lysates when treated with SMP4003 (related to data shown in Figure 3B).

Panel E: Anti-β-actin antibody with CT26 tumor lysates when treated with SMP4003 (related to data shown in Figure 3B).

Panel F: Anti-PE antibody with CT26 tumor lysates when treated with SMP4004 (related to data shown in Figure S6).

Panel G: Anti-β-actin antibody with CT26 tumor lysates when treated with SMP4004 (related to data shown in Figure S6).

**Supplemental references**

1. Bradford, M.M. (1976). A rapid and sensitive method for the quantitation of microgram quantities of protein utilizing the principle of protein-dye binding. Anal. Biochem. *72*, 248–254.
